# T cell exhaustion in parasitic infections

**DOI:** 10.3389/fimmu.2026.1910471

**Published:** 2026-09-04

**Authors:** Ge Gao, Hao Wang, Rafeezul Mohamed, Yinming Liang

**Affiliations:** 1 School of Nursing, Henan Medical University, Xinxiang, China; 2 Pusat Kanser Tun Abdullah Ahmad Badawi, Universiti Sains Malaysia, Pulau Pinang, Malaysia; 3 Department of Anesthesiology and Perioperative Medicine, The Third Affiliated Hospital Of Henan Medical University, Henan Medical University, Xinxiang, China; 4 Laboratory of Genetic Regulators in the Immune System, Henan Collaborative Innovation Center of Molecular Diagnosis and Laboratory Medicine, School of Medical Technology, Henan Medical University, Xinxiang, China

**Keywords:** immunosuppressive microenvironment, inhibitory receptors, parasitic infection, T cell exhaustion, Th1/Th2 response

## Abstract

T cell exhaustion is a state of progressive functional impairment that occurs upon persistent antigenic stimulation and is a key mechanism by which chronic parasitic infections evade host immunity. As research into T cell exhaustion continues to deepen, immunotherapies targeting exhausted T cells have emerged as a prominent area of interest, particularly in cancers and chronic viral infections. However, T cell exhaustion caused by parasitic infections exhibits significant differences from classic T cell exhaustion observed in chronic viral infections, and understanding how parasites with their complex life cycles and unique immune evasion strategies, drive or modulate T cell exhaustion will be essential for developing targeted therapeutic approaches for diverse diseases. Therefore, this review summarizes the current understanding of mechanisms and research progress regarding T cell exhaustion in the context of chronic infections, with a specific focus on parasitic infections and their different developmental changes, meanwhile raises several key questions which still remain unanswered, aiming to provide insights and references for immunotherapy development in related diseases.

## 1 Introduction

T cell exhaustion is a state of progressive functional impairment that occurs upon persistent antigenic stimulation and is a key mechanism by which chronic parasitic infections evade host immunity. The first description of T cell exhaustion dates back to 1998, in the classic study of a mouse model of chronic lymphocytic choriomeningitis virus (LCMV) (1, 2). Since then, T cell exhaustion has been demonstrated in various chronic infections and cancers, as well as in animal models (3–9). In 2025, a T-cell nomenclature guideline, co-authored by 72 leading immunologists worldwide, summarizes different subsets of exhausted T cells (10): Progenitor or precursor exhausted T cells (TPEX) possess self-renewal capacity, can differentiate into more terminally exhausted subsets; Intermediate exhausted T cells (TEX-int) and effector-like exhausted T cells (TEX-eff) are in a transitional state, which derived from progenitor cells; Terminally differentiated exhausted T cells (TEX-term), with low proliferative capacity, have weakened effector functions but still retain limited cytotoxicity.

As research into T cell exhaustion continues to deepen, immunotherapies targeting exhausted T cells have emerged as a prominent area of interest. This review summarizes the current understanding of mechanisms and research progress regarding T cell exhaustion in the context of chronic infections, with a specific focus on parasitic infections, aiming to provide insights and references for immunotherapy development in related diseases.

## 2 Characteristics of T cell exhaustion

Exhausted T cells (Tex) represents a distinct population, with characteristics that are quite different from those of Tmem and Teff, such as progressive functional loss, upregulation of multiple inhibitory receptors, alterations in epigenetic and transcriptional profiles, and changes in metabolic activity. These fundamental mechanisms, initially elucidated in viral infection models, are now recognized as broadly applicable to chronic parasitic infections, although with distinct features that will be discussed in detail in Section 3.

### 2.1 Progressive loss of effector function

**Figure 1:**
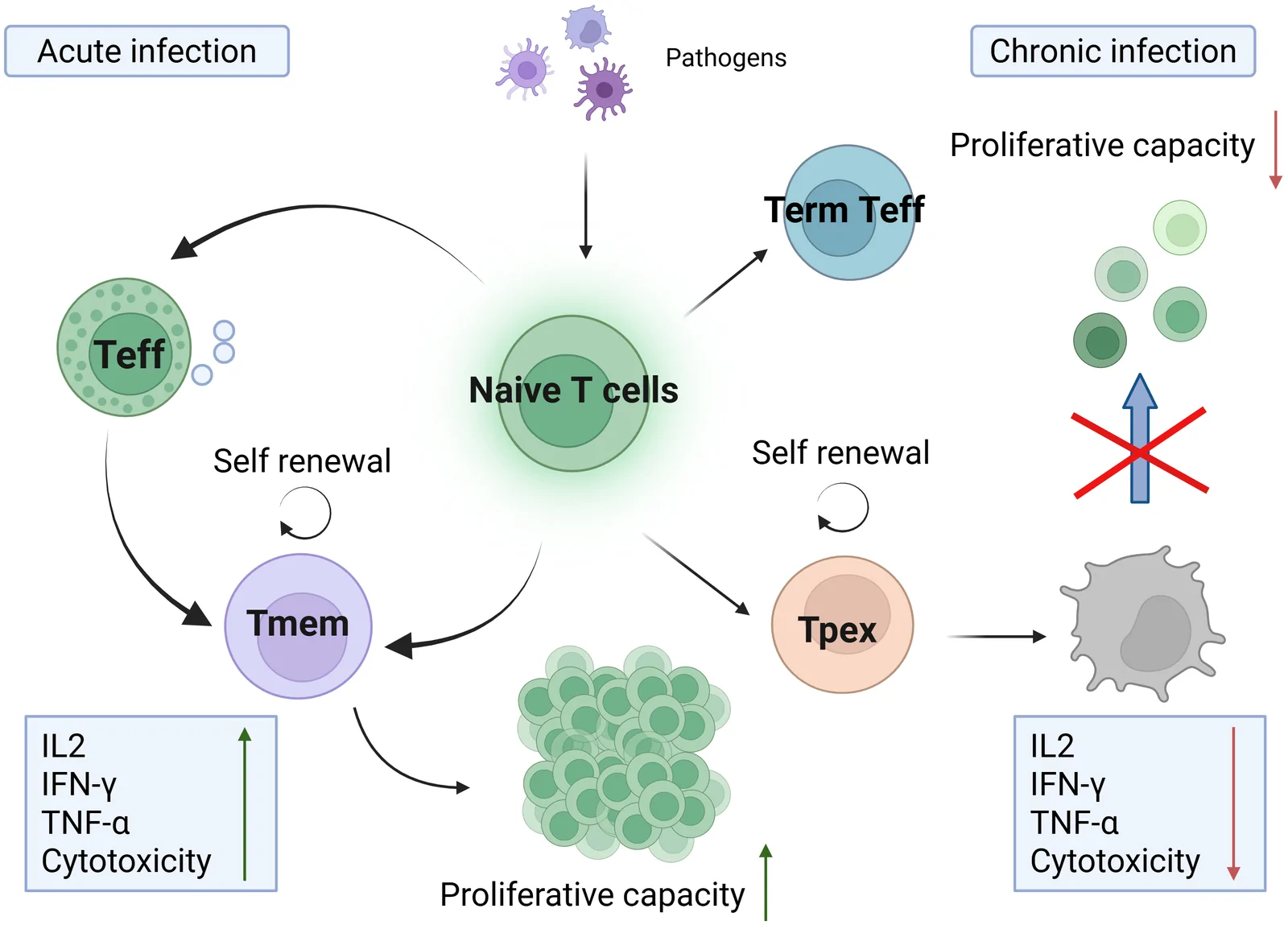
Features of T-cell exhaustion in chronic infection. Antigen, costimulation, and inflammation activate naive T cells during acute infection, leading to development of Teff cells or long-lived Tmem cells. A minority of functional Teff cells may develop into highly polyfunctional Tmem cells capable of coproducing multiple cytokines, exerting cytolytic activity, and proliferating rapidly after antigen clearance. However, during chronic infection, prolonged antigen exposure triggers an alternative T cell differentiation program. Early in chronic infection, some naive T cells differentiate into terminal effector-like T cells that undergo rapid decline. Concurrently, other naive T cells differentiate into progenitor exhausted T cells (Tpex), which possess self-renewal capacity and eventually, irreversibly, become terminally exhausted T cells (Tex). Throughout this process, T cells progressively lose effector functions.

While exhaustion encompasses multiple features, its defining characteristic is a progressive loss of functionality among fully activated CD8^+^ T cells. This is a dynamic, gradual, and multi-layered process, not a simple on/off switch of function. Rather, it is the orderly and phased loss of key effector functions by T cells under continuous antigen stimulation (11, 12). Subsequent studies have demonstrated that CD8+ T cells progressively lose functions in a hierarchical manner during exhaustion (11–13). Reduction of IL-2 and TNF-α production occurs first, accompanied by impaired cell-mediated cytotoxicity, decreased proliferative capacity and the upregulation of inhibitory receptors (12). Nonetheless, CD8+ T cells might still be able to generate IFN-γ at this point. At more severe stages of exhaustion, IFN-γ production becomes abnormal. T cells have evolved from being able to produce multiple effector molecules simultaneously to being single-functioning cells that can only produce a few molecules. Ultimately, all effector functions are completely lost (14–16), as illustrated in **Figure 1**.

The functional status of antigen-specific CD8+ T cells in chronic infections spans a spectrum: they may be fully functional, partially exhausted, completely exhausted, or physically deleted, depending on the pathogen, the specific epitope, antigen load, and infection duration. Higher viral loads, more antigenic epitopes presented, longer infection duration, and lack of CD4+ T cell help all correlate with more severe exhaustion (12, 13, 17). This hierarchical loss of function suggests that therapeutic interventions might be most effective when applied before irreversible dysfunction occurs.

### 2.2 Sustained expression of inhibitory receptors

Inhibitory receptors (IRs) play a negative regulatory role in controlling immune reactivity and preventing immunopathology, thereby protecting the host from dangerous inflammatory responses (18). During acute infections, IR expression transiently increases to modulate T cell activity and declines after antigen clearance (19, 20). In contrast, during cancer and chronic infections, IR expression remains persistently high. This sustained and/or elevated expression of multiple IRs is a key feature of both CD8+ and CD4+ T cell exhaustion. Importantly, this characteristic suggests that exhausted T cells may be reversible through appropriate pathway targeting, which is a concept confirmed by numerous studies and now a cornerstone of cancer immunotherapy (21–23). The sustained IR expression represents both a marker of exhaustion and an actionable therapeutic target. The expression of inhibitory receptors on exhausted T cells in chronic parasitic infections will be further discussed in Section 3.

### 2.3 Altered memory maintenance

Following acute infection clearance, generated memory T cells acquire the capacity for stable, cytokine-driven self-renewal mediated by IL-7 and IL-15, enabling long-term maintenance without antigen stimulation (24). However, due to lower CD127 (IL-7Rα), a component of the IL-7 receptor, and CD122 (IL-2Rβ) expression levels, interruption of transmission in critical signal paths and locking of epigenetics, virus-specific exhausted T cells cannot achieve homeostatic proliferation through IL-7 and IL-15 during chronic infection (25, 26). An early study by Shin et al. revealed that these CD8+ T cells did not acquire cardinal memory T cell properties. They were unable to undergo homeostatic proliferation, exhibited decreased responsiveness to IL-7/IL-15, and blocking IL-7 signaling in IL-15 knockout mice had minimal effect on CD8+ T cell numbers during chronic infection, demonstrating that IL-7/IL-15 signaling is not essential (27).

This creates an apparent paradox: these cells persist long-term despite lacking cytokine-driven maintenance. The resolution lies in understanding that exhausted T cell maintenance depends on continuous TCR stimulation (27, 28). The study by Shin et al. revealed that virus-specific CD8+ T cells could not be maintained long-term in the absence of cognate antigenic peptide. Even within the same chronic infection environment, only T cells capable of recognizing persistent antigens remained stable. Therefore, during chronic infection, T cell maintenance depends on continuous antigen stimulation rather than solely on cytokines or inflammatory environment (27).

This has also been confirmed in parasitic infections. A study about *Trypanosoma cruzi* infection revealed that exhausted T cells only appeared after long-term chronic infection (>2 years), suggesting that continuous local antigen stimulation is a key factor driving T cell exhaustion (29). Furthermore, a recent study shows that epigenetic remodeling induced by the transcription factor TOX locks cells into an exhausted state. Chronic antigen stimulation is also a necessary condition for TOX induction (30). Memory T cells, however, are not dependent on TOX, directly explaining the different molecular basis of their long-term survival mechanisms. This antigen-dependent persistence fundamentally distinguishes exhausted T cells from memory T cells and explains why antigen clearance is necessary for recovery from exhaustion.

### 2.4 Metabolic changes

T cell metabolism exhibits high dynamic plasticity, enabling rapid reprogramming of metabolic pathways to support diverse functional needs based on differentiation state and microenvironmental signals (31). For example, naive T cells have low energy requirements due to their quiescent state; their primary metabolic pathway is oxidative phosphorylation (OXPHOS), supplemented by low-level fatty acid oxidation (FAO) and glycolysis. By efficiently producing ATP, they maintain long-term survival and immune surveillance functions while preparing for rapid activation (32–34). Upon activation for growth, proliferation, and clonal expansion, cells exit this quiescent state and activate aerobic glycolysis and glutamine metabolism via the PI3K/Akt/mTOR pathway, supporting cell growth and proliferation (35, 36).

However, exhausted CD8+ T cells exhibit dramatic alterations in metabolic and bioenergetic pathways. Exhausted CD8+ T cells downregulate molecules involved in the citric acid cycle and energy metabolism, and inhibit both mitochondrial respiration and aerobic glycolysis, suggesting metabolic deficiencies evident in gene expression profiles (37–40). For example, the inhibitory receptor CD244, in the *Echinococcus multilocularis* infection, promotes CD8+ T cell exhaustion by inducing mitochondrial dysfunction, such as significantly increasing the reactive oxygen species levels in CD8+ T cells and reducing ATP-linked respiration (41). It has also been confirmed in CD4+ T cell exhaustion. During malaria infection, exhausted CD4+ T cells exhibit decreased mTOR activity and reduced glycolytic capacity, and this metabolic defect is associated with loss of T-bet expression (42). Leishmania can also evade immune clearance and shape an immunosuppressive microenvironment favorable for its survival by reprogramming key metabolic pathways in macrophages, dendritic cells, and T cells (43). Additionally, metabolic stress can induce endoplasmic reticulum (ER) stress responses, which may reinforce T cell exhaustion (44, 45).

These metabolic defects may partially result from inhibitory receptor signaling (35). Several studies have shown a correlation between inhibitory receptors and metabolism (46, 47), which raises an intriguing possibility that combining immunotherapy with metabolic modulators such as glycolysis inhibitors could enhance therapeutic efficacy.

### 2.5 Epigenetic landscape of exhausted T cells

Regarding exhausted T cells, the initial view was that they share characteristics with terminally differentiated T cells, suggesting a connection in their developmental pathways (48, 49). However, subsequent studies have confirmed that Tex cells originate from the same progenitor cells as Tmem cells, rather than from terminal effector T cells (50). It is now understood that the T cell exhaustion program occurs simultaneously with normal T cell differentiation. An alternative viewpoint holds that cells at any differentiation stage can initiate the exhaustion process (51).

**Figure 2:**
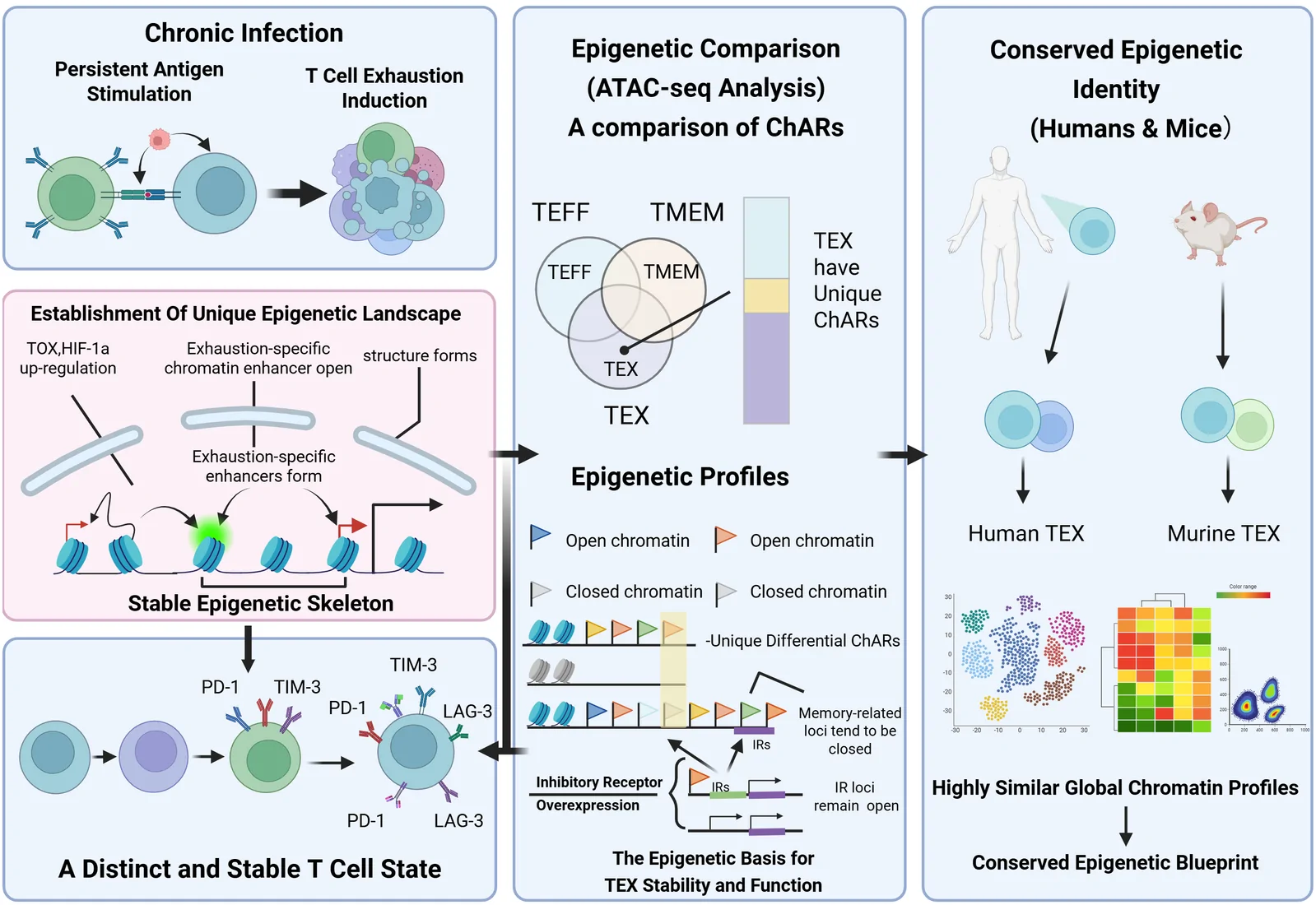
Epigenetic landscape of exhausted T cells. Exhausted T cells constitute a unique cell lineage, quite different from effector and memory cells. In exhausted T cells, exhaustion-specific super-enhancers form at some key transcription factor loci (such as TOX and HIF-1α) and the entire chromatin landscape's skeleton is locked in an exhaustion state. Even after antigen clearance, these exhaustion features still remain, becoming epistemological scarring. In both humans and mice, exhausted T cells exhibited highly similar global chromatin profiles, which means exhausted CD8+ T cells represent a distinct and stable T cell state generated under particular conditions.

Epigenetic studies have revealed that exhausted T cells constitute a unique cell lineage (52). Exhausted CD8+ T cells exhibit distinctly different characteristics from both effector and memory cells (52, 53). ATAC-seq analysis revealed marked differences in chromatin-accessible regions (ChARs) between exhausted CD8+ T cells and functional effector or memory CD8+ T cells (53). Nearly half (44.48%) of all ChARs in exhausted T cells differed from those in functional cells, a magnitude of difference comparable to that between distinct hematopoietic lineages (54). An early study also demonstrated that T cells exhibit specific DNA methylation characteristics in cases of parasitic infections (55). Together, these studies demonstrate that exhausted CD8+ T cells represent a distinct and stable T cell state generated under particular conditions, characterized not only by high inhibitory receptor expression but by a unique epigenetic identity, as illustrated in **Figure 2**.

In 2021, Yates et al. proposed the concept of “epistemological scarring” (56). Episemological scarring refers to a unique and stable epigenetic landscape established by the T-cell exhaustion program in chronic infection or tumors, which cannot be completely reversed even after antigen clearance (56–59). These residual exhaustion features persistently limit the functional recovery and memory potential of T cells. When antigens are cleared, only a small percentage of cells (approximately 10%) show transcriptional changes accompanied by alterations in chromatin accessibility, which means that although the transcriptional activity of some genes can be restored, the entire chromatin landscape’s skeleton is locked in an exhaustion state, making it impossible to regain the epigenetic features of memory T cells (57). The concept of episemological scarring provides direct evidence for the long-term persistence and irreversibility of exhausted T cells.

In chronic parasitic infections, the epigenetic landscape of exhausted T cells undergoes a fundamental and highly stable remodeling, endowing them with a unique, heritable epigenetic identity. DiNardo et al. found that co-infection with helminths (*Schistosoma* and *Ascaris*) induces differential DNA methylation of 751 genes in CD4+ T cells (60). These epigenetic alterations persist long-term and suppress the host’s Th1-type protective immunity against Mycobacterium tuberculosis. After successful deworming, these changes persisted for at least six months in individuals infected with *Schistosoma haematobium*, directly demonstrating that helminth infection can induce durable DNA methylation changes in host CD4^+^ T cells. Meanwhile, in Leishmania infection, exhausted and senescent T cells co-exist in the circulation and skin of patients. Moreover, CD8^+^ tissue-resident T cells in skin lesions exhibit three distinct exhaustion stages of progenitor, transitional, and terminal, providing cell-biological evidence for the existence of epigenetic scarring (61).

Therefore, this epigenetic reprogramming explains the relative stability of the exhausted state and why reversal often requires more than simply removing the driving antigen or blocking inhibitory pathways.

## 3 T cell exhaustion in parasitic infections

Many studies have demonstrated that T cell exhaustion occurs in parasitic diseases. However, T cell exhaustion caused by parasitic infections exhibits significant differences from classic T cell exhaustion observed in chronic viral infections. These differences likely reflect the distinct evolutionary strategies of parasites, which often favor chronic coexistence rather than rapid clearance, and the corresponding immune responses that balance control with tissue protection. Therefore, deep understanding of the characteristics and mechanisms of T cell exhaustion in parasitic infections is critical for designing effective immunotherapeutic strategies.

### 3.1 Th1/Th2 polarization and exhaustion

Many chronic infections and cancers share a common characteristic: persistent T cell exposure to antigens. Persistent antigen exposure is one of the crucial factors triggering T cell exhaustion (62). Increased antigen burden and extended periods of antigen stimulation both lead to more profound T cell exhaustion (63).Wherry et al. were the first to systematically describe the hierarchical nature of progressive CD8+ T cell exhaustion caused by persistent viral infection in an LCMV model, pointing out that this dysfunction is directly related to viral load (antigen load) (12). However, it is different in parasitic infections.

In parasitic infections, intracellular infections are primarily characterized by Th1-type immune responses, similar to viral infections. For example, leishmaniasis caused by *Leishmania* relies on Th1-type immune responses, activating macrophages through IFN-γ secretion to produce reactive nitrogen intermediates that kill intracellular protozoa (64, 65). Similarly, in *Toxoplasma gondii* infection, tachyzoites activate the neutrophil-macrophage axis after foodborne intestinal invasion, initiating IL-12/IFN-γ-mediated Th1-type immune responses (66). This response directly eliminates infected cells and promotes phagocyte activation to clear intracellular parasites. However, even with robust T cell responses, achieving complete pathogen eradication is uncommon, ultimately leading to chronic infection. While during chronic parasitic infections, there is numerous research data confirm the existence of T cell exhaustion, which, consistent with other chronic infections, contributes to deterioration of host infection control (67–70).

However, T cell exhaustion in parasite infection models differs considerably from that in viral infections. This difference stems from the distinct biological characteristics, co-evolutionary strategies with the host, and types of immune responses elicited by the two types of pathogens. Chronic viral infections are often accompanied by persistent high levels of viremia, with high and concentrated antigen loads, while chronic parasitic infections are typically characterized by lower pathogen burden that remains largely confined to specific tissues, suggesting that high persistent antigen levels are not the primary determinant of CD8+ T cell exhaustion in parasitic models. For instance, in acute *T. gondii* infection, parasites typically differentiate into semidormant bradyzoites and form tissue cysts under adaptive immunity influence (71). However, after using *T. gondii* ME49 strain cysts to infect C57Bl/6 mice, T cell exhaustion still occurs and leads to mouse mortality 7 weeks later (72). Viruses provide a relatively pure chronic antigen stimulation model, making the mechanisms of T cell exhaustion in viral models the most thoroughly and classically studied, and their characteristics are very clearly manifested in viral systems. Chronic parasitic infections, however, are more complex, with unique factors contributing to T cell exhaustion in addition to persistent antigen stimulation.

Another instructive example is Chagas disease caused by *Trypanosoma cruzi*. Bustamante et al. reported that in a murine model of *T. cruzi* infection, sustained exposure to a persistent pathogen did not lead to the development of overt exhaustion in pathogen-specific T cells, contradicting the conventional view that exhausted T cells depend on antigen persistence (73). However, although Bustamante observed no evidence of T cell exhaustion in 2-year-long T. cruzi infections in mice, signs of functional impairment consistent with immune exhaustion have been observed in humans after infection for more than 20 years (73, 74). Several earlier studies also reported T cell exhaustion in chronic Chagas disease. This suggests two distinct temporal modes of exhaustion development: the classic LCMV model where persistent high antigen load drives rapid exhaustion, and the T. cruzi model where low antigen load in nonprofessional antigen-presenting cells drives exhaustion only after extremely prolonged (decades-long) exposure.

In helminth infections, Th2 cells and the immune responses they mediate play central roles. Since large worms are difficult to directly engulf and eliminate, Th2 immune responses primarily aim to expel worms and repair tissues. In intestinal worm infections, type 2 innate lymphoid cells (ILC2s) respond rapidly in early infection stages, providing first-line defense, while Th2 cells are more crucial for establishing adaptive immunity during long-term chronic infections (75). However, although Th2 immune responses help hosts expel parasites and repair tissue damage, they can also lead to immunopathology in chronic infections (76). In studies of Schistosoma infection, Th1-type immune responses were observed in early infection stages. However, as eggs deposited in the liver, the response shifted to a Th2-type immune response driven by IL-4/IL-13. Although this response temporarily alleviated acute damage, it ultimately led to liver fibrosis (77–79). Thus, the Th2 effect is a double-edged sword: a delicate balance must be struck between expelling parasites and avoiding excessive tissue damage.

**Figure 3:**
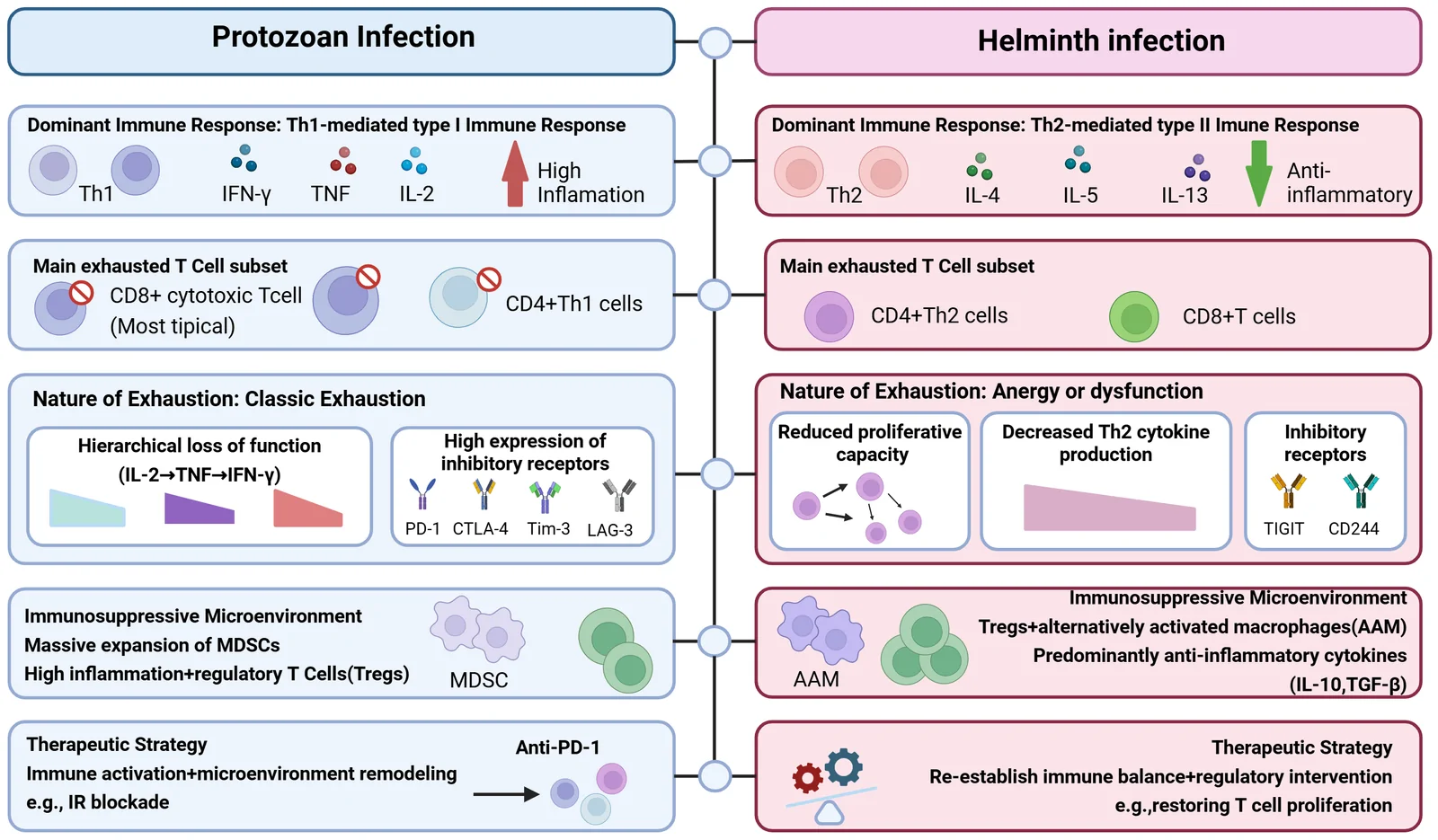
T cell rxhaustion: prozozoan infection vs. helminth infection. The profiles of T cell exhaustion in helminth infections are considerably different from those in protozoan infections. T cell exhaustion induced by protozoan infections represents the classic form of T cell exhaustion, similar to that observed in viral infections. In contrast, T cell exhaustion caused by helminths is more characterized by functional anergy or dysfunction. In addition, they also differ in many other aspects, including dominant immune responses, main exhausted T cell subsets, immunosuppressive microenvironment, and therapeutic strategies.

It is well established that CD8+ T cells undergo exhaustion program in chronic Th1 infections, and other studies provide evidence for Th2 cell-intrinsic dysfunction development. **Figure 3** illustrates the differences in T cell exhaustion between helminth and protozoan infections. An early study by Taylor et al. pioneered the understanding of the downregulation and mechanisms of Th2 cell function in chronic *Schistosoma mansoni* infection. This study indicated that the function of Th2 cells significantly declined during chronic schistosomiasis infection and this Th2 hyporesponsiveness was intrinsic to the cells, independent of inhibitory signals provided by antigen-dependent antigens (APCs) and unrelated to classical inhibitory receptors. Gene related to anergy in lymphocytes (GRAIL) upregulation is a key mechanism by which Th2 cells enter a hyporesponsive state after repeated antigen stimulation, representing a unique intrinsic regulatory pathway specific to Th2 cells (80). Another study by van et al. also confirmed the intrinsic functional hyporesponsiveness of Th2 cells in the infection of filarial nematode. In chronic *L. sigmodontis* infection, Th2 cell function is progressively lost, mainly in terms of proliferative capacity and cytokine production, while PD-1 expression significantly increases with the progression of infection. PD-1 blockade restores Th2 cell function in the long term, rather than increasing their number. More importantly, PD-1 regulated immune responses through PD-L2, contrasting with the PD-L1-dependent pathway observed in viral and protozoan infections (81). This PD-L2 dependence represents a distinct regulatory mechanism in Th2 contexts and suggests that immunotherapies effective in Th1-dominated settings may not directly translate to some helminth infections. Nevertheless, “Th2 cell exhaustion” remains a concept without clear definition and broad consensus, unlike CD8+ T cell exhaustion in chronic viral infection. Therefore, deep understanding of T cell exhaustion mechanisms in Th2 contexts is critical.

### 3.2 Inhibitory receptors in parasitic infections

Inhibitory receptors negatively regulate autoreactivity and immunopathology. As in tumors and chronic viral infections, the sustained high expression of multiple inhibitory receptors is also a core hallmark of T cell exhaustion in parasitic infections. This has been confirmed in various parasitic infections, including malaria, *Leishmania*, *Trypanosoma cruzi*, *Toxoplasma*, *Schistosoma*, and *Echinococcus*.

In central nervous system (CNS) parasitic infections represented by neurotoxoplasmosis caused by *Toxoplasma gondii* and neurocysticercosis caused by *Taenia solium*, both infections lead to high expression of multiple inhibitory receptors on T cells, such as PD-1, TIM-3, LAG-3, KLRG1, and CTLA-4 (82). And anti-PD-1 antibody has been shown in mouse models of neurotoxoplasmosis to reduce cyst burden and promote brain lymphocyte infiltration. Moreover, anti-PD-1 immunotherapy can effectively limit lesion progression and reduce parasite burden in the mouse model of chronic cutaneous leishmaniasis by remodeling the exhausted T cells (Tex) subpopulation structure, particularly by enhancing CXCR5+ Tex cells with progenitor/stem-like characteristics (83). This provides a foundation for clinical translation of PD-1 blockade to regulate Tex cell differentiation for the treatment of human chronic leishmaniasis. Similarly in patients with chronic Chagas disease, co-expression and upregulation of multiple inhibitory receptors also have been observed, and CTLA-4 expression on peripheral T cells and in myocardial tissue of infected individuals suggests its possible involvement in cardiac pathology (84). Another study found that in Schistosoma mansoni infection, the inhibitory receptor CD200R and its ligand CD200 may constitute a novel immunosuppressive axis involved in the exhaustion program, indicating its therapeutic potential as a new immune checkpoint (85).

Additionally, plasmodium infection can drive CD4+ T cell exhaustion involving PD-1 and CTLA-4 (42). Interestingly, however, some studies have found that although malaria-induced CD8+ T cells upregulate multiple co-inhibitory molecules such as LAG-3 and PD-1, they exhibit enhanced functionality rather than functional exhaustion (86). This unique pattern of inhibitory receptor expression clearly demonstrates that upregulation of inhibitory receptors does not necessarily lead to functional loss. Only under chronic, persistent antigen stimulation does this transient expression evolve into sustained high expression, driving genuine T cell exhaustion.

In summary, exhausted T cells display a pattern of co-expression of multiple inhibitory receptors in chronic parasitic infections. These molecules act synergistically through distinct signaling pathways to collectively construct a potent immunosuppressive network. Therefore, blockade of a single target has limited efficacy. For example, in *Echinococcus multilocularis*-infected mice, PD-1 pathway blockade alone fails to restore the function of exhausted T cells (87). This contrasts with certain viral infections such as LCMV or HIV, where PD-1/PD-L1 blockade alone can more robustly restore T cell function (88, 89), highlighting the greater complexity of the immunosuppressive network in parasitic infections and suggesting that therapeutic strategies developed in viral systems may not be directly translatable. The differential efficacy of blocking various inhibitory pathways suggests that exhaustion is maintained through non-redundant mechanisms that may require combination approaches for optimal reversal.

### 3.3 Costimulatory signals in parasitic infections

Costimulatory molecules expressed by CD8+ T cells, including CD28, CD27, 4-1BB, and OX40, are essential for orchestrating effective T cell responses. They play a critical role in promoting T cell activation, supporting clonal expansion, ensuring cell survival, and establishing long-term immunological memory (90, 91). For example, CD28, the most important co-stimulatory molecules, provides the second signal required for activation, preventing T cells from entering a dysfunctional state (92–94). In the absence of costimulatory signals, antigen stimulation is believed to induce tolerance or clonal deletion (95).

An imbalance in costimulatory signals, whether through excessive dampening or inappropriate enhancement, can promote T cell exhaustion. In a mouse malaria model, blockade of the B7/CD28 pathway leads to an imbalance between Th1 and Th2 responses (96). And the study by Ndlovu et al. further demonstrated that CD28 knockout mice infected with intestinal nematodes exhibited impaired Th2 responses and failed to effectively clear the parasites, suggesting that sustained CD28 signaling is critical for maintaining memory T cell function and preventing exhaustion (97). In chronic parasitic infections, downregulation or impaired signaling of CD28 may promote T cell exhaustion. Furthermore, Kamphorst et al. investigated the role of CD28 in rescuing exhausted CD8+ T cells after PD-1 treatment and found that CD28 was essential for effective PD-1 blockade therapy, as conditional CD28 deletion diminished PD-1 pathway blockade efficacy (98).

Thus, in addition to inhibitory receptors, costimulatory receptors also contribute to T cell exhaustion. CD8+ T cell exhaustion is characterized by high coinhibitory receptor expression and is promoted both by antigen persistence and by insufficient costimulation. This dual requirement, reducing inhibitory signals while maintaining costimulatory input, has important implications for combination immunotherapy design.

### 3.4 Transcriptional control in parasitic infections

In chronic parasitic infections, T cell fate, whether to become memory or exhausted, is finely regulated by a sophisticated network of transcription factors. Persistent parasite antigens remodel the intrinsic programming of T cells, shifting them from a protective effector state to a functionally impaired exhausted state.

TOX is one of the master switches controlling the exhaustion program. During chronic parasitic infections, high levels of TOX expression on CD8+ T cells is required to drive and maintain the exhaustion phenotype. TOX reprograms T cells at both the epigenetic and transcriptional levels, establishing and maintaining an exhaustion specific gene expression profile that ultimately leads to loss of T cell function (56, 99, 100). This process also locks the cells into the exhausted state, making it difficult to reverse. TOX establishes exhaustion-specific super enhancers and open chromatin regions at key DNA gene loci, leading to the formation of epigenetic scars, explaining how the exhausted state becomes epigenetically fixed (99, 101). Even with downregulation of TOX, epigenetic scars still persist (56, 57).

Beyond this core switch, other transcription factors also finely regulate T cell fate through distinct pathways. One example is Id2. In chronic central nervous system (CNS) *Toxoplasma gondii* infection, the expression level of Id2 is a key determinant of whether CD8+ tissue-resident memory T (Trm) cells differentiate into a potent effector state or a functionally exhausted state. Id2 deficiency drives T cells toward exhaustion (PD-1+ TOX+), whereas Id2 overexpression effectively suppresses exhaustion, suggesting that Id2 might serve as a therapeutic target to reverse T cell exhaustion by enhancing Id2 expression (102).

Moreover, signaling-related transcription factors, such as NFAT1, a member of the NFAT family, and T-bet, link external stimulation to cellular metabolic and functional changes. In chronic malaria models, NFAT1 has been shown to mediate CD4+ T cell exhaustion by inhibiting effector gene expression, and NFAT1 deficiency enables CD4+ T cells to resist exhaustion and maintain robust effector functions (103). Meanwhile, exhausted CD4+ T cells also exhibit decreased mTORC1 activity and reduced glycolytic metabolism, which correlates with decreased T-bet expression (42). In *Toxoplasma gondii* infection, reduced expression of both T-bet and Eomes is a hallmark of loss of polyfunctionality (104).

Transcription factor Blimp-1 is closely associated with CD4+ T cell exhaustion. In chronic *Toxoplasma gondii* infection, intrinsic Blimp-1 expression in CD4+ T cells leads to progressive exhaustion, which in turn causes CD8+ T cell dysfunction and decreased pathogen control (105). Khan et al. further proposed a novel mechanism by which miR146a upregulates Blimp-1 expression through targeting TRAF6, leading to CD4+ T cell exhaustion. Conditional knockout of Blimp-1 in CD4+ T cells restores CD4+ T cell function, reverses CD8+ T cell exhaustion, and prevents reactivation of latent infection, highlighting the interconnected regulation of exhaustion across T cell subsets (106).

These findings identify multiple transcription factors as potential targets for reversing T cell exhaustion and improving immune control in chronic parasitic diseases.

### 3.5 Regulatory T cells in parasitic infections

Regulatory T cells (Tregs) are a CD4+ T cell subset whose main functions include suppressing allergies, preventing autoimmune diseases, and maintaining self-tolerance (107, 108). Unlike chronic viral infections where CD8+ T cells continuously attempt to eliminate infected cells, the aim of immune responses in chronic parasitic infections, dominated by Th1 (protozoa) or Th2 (helminths) responses, is not to kill but to control, and is therefore often accompanied by large numbers of Tregs and strong immunomodulation (107, 109, 110). This is because parasites have complex structures, long lifecycles, and are adept at immune evasion. Immune responses, especially Th2 responses against helminths, often focus on expelling or encapsulating parasites (e.g., through granuloma formation) rather than complete eradication. Consequently, responses are often chronic and accompanied by strong regulatory mechanisms (such as Tregs and IL-10) to prevent excessive immunopathological damage, which also creates conditions for long-term parasite coexistence (111).

A study on the intestinal helminth parasite *Trichuris muris* confirmed that parasites may drive Tregs to specifically limit Th2 immunity to prolong their survival, rather than inducing broad, non-specific immune dampening. The study also identified that Treg depletion outcomes are temporal: early Treg targeting can accelerate parasite clearance beneficial to the host, but once infection is established, Treg depletion actually enhances parasite burden and immune pathology (112).

Chronic Schistosoma infection has been shown to be associated with general T cell hyporesponsiveness. Schmiedel et al. assessed the frequency and functional activity of Treg cells in human *Schistosoma haematobium* infection and provided evidence that following *S. haematobium* infection, the frequency of CD4^+^CD25^hi^ FOXP3^+^ Treg cells significantly increased. After praziquantel treatment, the Treg frequency decreased, but its inhibitory effect on cytokines remained, while its inhibitory effect on proliferation weakened, which suggests that even after the parasite is cleared, the immune regulatory network may continue to influence subsequent immune responses (113). In chronic Schistosoma infection, Treg cells are key executors of systemic immunosuppression (T cell hyporesponsiveness), and their function is persistent. In *T. cruzi* infection, Treg cells play a role during initial stages, restraining CD8+ T cell response magnitude and parasite control (114).

However, previous reports on Treg cell roles in parasitic infection have been somewhat controversial, with some studies describing beneficial impacts. For example, a study by González et al. suggested that Treg cells show reduced proliferation rates and progressively decrease in frequency during *T. cruzi* infection compared to effector T cells, leading to worsened myocarditis and shortened survival time (115). Thus therapies aimed at specifically boosting Treg cells might represent new opportunities for Chagas disease treatment. In malaria infection, Tregs prevent the development of experimental cerebral malaria. Mice lacking Tregs, while better able to control the parasite, died from brain immunopathology (116, 117). Similarly, in leishmaniasis infection, Tregs limit local inflammation and tissue necrosis in cutaneous leishmaniasis (118, 119). Although these Treg cells also inhibit complete parasite clearance, they protect the integrity of the tissue. The apparent paradox that Tregs both promoting chronic infection and protecting against immunopathology highlights the context-dependent nature of regulatory responses.

A further layer of complexity comes from studies of PD-1-deficient mice and blockade of the PD-1 pathway during parasitic infections. In a Schistosoma japonicum infection model, Lu et al. found that loss of PD-1 markedly increased T cell infiltration in the liver, but this was accompanied by excessive Treg-mediated immunosuppression, which paradoxically antagonized effective anti-parasite T cell immunity (120). The PD-1/PD-L1 pathway does not simply promote T cell exhaustion; it also plays a context-dependent role in restraining regulatory T cell activity. Perry et al. demonstrated that PD-L1–PD-1 interactions limit effector regulatory T cell (eTreg) populations at homeostasis, and during *Toxoplasma gondii* infection, early interferon-γ upregulates PD-L1 on myeloid cells, which is associated with reduced eTreg populations (121).

**Figures 4:**
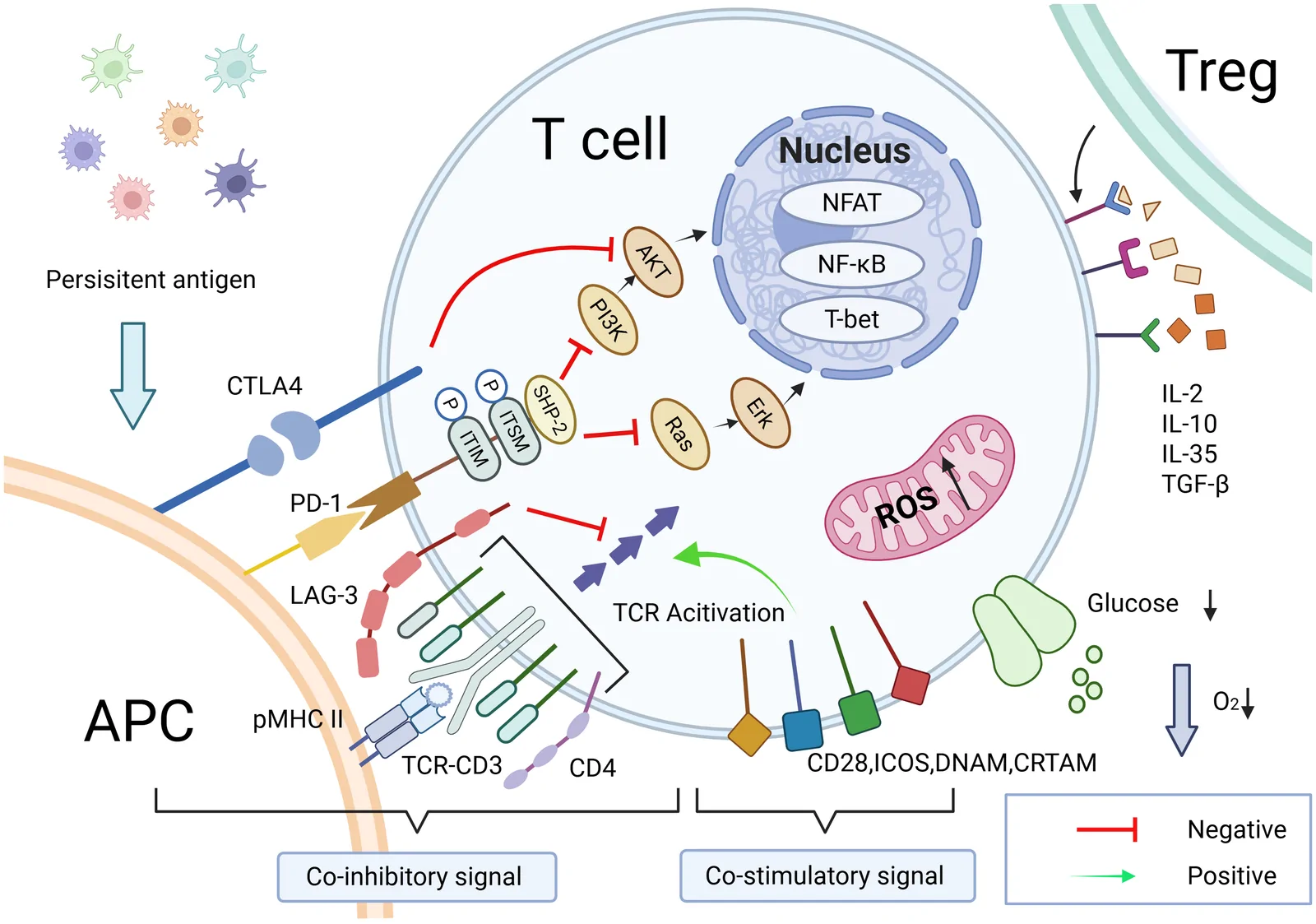
Mechanisms of T‑cell exhaustion. The combined effect of multiple factors leads to T cell exhaustion. Persistent antigen stimulation drives T cell hyperactivation, eventually leading to sustained co-expression of multiple inhibitory receptors on T cells and their ligands on antigen-presenting cells (APCs). High coinhibitory receptor expression and lack of costimulatory signals prevent optimal T cell effector responses. Other factors, including cytokines (IL-2, IL-10, IL-35, TGF-β) and microenvironmental changes (reduced nutrient availability, hypoxia), also contribute to T cell exhaustion.

Therefore, Treg cell modulation during chronic infection should be carefully examined, and more detailed investigations are necessary to fully understand Treg cell roles. **Figure 4** illustrates the mechanisms of T cell exhaustion.

### 3.6 Hypoxia-driven exhaustion in parasitic infections

In chronic parasitic infections, there is a direct causal relationship between hypoxia and T cell exhaustion. Histopathological changes caused by parasitic infections, such as granulomas and fibrosis, damage normal tissue vascular structures, severely hindering blood flow and oxygen delivery, creating a hypoxic microenvironment at infection sites. Hypoxia, in turn, affects T cell function through metabolic reprogramming, promoting exhaustion. A study on alveolar echinococcosis (AE) caused by *Echinococcus multilocularis* measured the oxygen consumption rate (OCR) of CD8+ T cells derived from mouse liver and spleen. Compared to control mice, CD8^+^ T cells from infected wild-type mice exhibited a significant reduction in ATP-linked mitochondrial respiration, confirming that T cell exhaustion in parasitic infections is accompanied by metabolic dysfunction, and metabolism is a core aspect of how hypoxia affects cell function (41).

Beside metabolic effects, hypoxia directly upregulates inhibitory receptors, promotes expression of exhaustion-related transcription factors such as TOX and NR4A, and inhibits production of effector cytokines including IFN-γ and TNF-α, thereby leading to loss of cell function and differentiation toward terminal exhaustion (122–125). Hypoxia also promotes Treg cell survival and function, induces immunosuppressive cell aggregation, and increases production of inhibitory metabolites such as adenosine through CD39/CD73 upregulation, creating an immunosuppressive microenvironment that further suppresses T cell function (126–129).

On the other hand, in hypoxic environments, cells stably express hypoxia-inducible factors (HIF), particularly hypoxia-inducible factor 1α (HIF-1α). In CD4^+^ and CD8^+^ T cells, HIF-1α functions downstream of both TCR and IL-2R signaling, facilitating the early glycolytic switch that occurs following T cell priming and inhibit mitochondrial oxidative phosphorylation (130). Hypoxia can drive T cell exhaustion at multiple levels through HIF-1α. Wei et al. first reported that HIF-1α is an upstream regulatory molecule of NFATC1/PD-1. *Plasmodium yoelii* infection induces anemia and hypoxia in mice, leading to stable HIF-1α expression. HIF-1α enters the nucleus and transcribes to activate NFATC1 expression, which then binds to the PD-1 promoter, initiating PD-1 expression on CD4^+^ T cells. PD-1 and NFATC1 expression are negatively correlated with erythrocyte density. *In vivo* experiments, treatment of infected mice with HIF-1α inhibitor YC-1 resulted in spleen shrinkage, and a simultaneous decrease in the expression of HIF-1α, NFATC1, and PD-1 in the spleen (131).

Additionally, a previous study by Hammami also provided evidence that the hypoxic microenvironment stabilizes HIF-1α, limiting protective Th1 response development, which benefited L. donovani survival and resulted in chronic infection establishment (132). In subsequent research, Hammami further confirmed that *Leishmania donovani* infection limited CD8+ T cell expansion by upregulating HIF-1α in dendritic cells and subsequently impairing DC functions (133).

Hypoxia-driven T cell exhaustion is a well-established mechanism in oncology and chronic viral infections, while direct research on its application in parasitic infections remains limited. Nevertheless, hypoxia likely represents a key bridge connecting chronic parasitic infection pathology with T cell dysfunction, and targeting hypoxia pathways may offer novel therapeutic opportunities. Given the prominent tissue hypoxia induced by parasite-driven pathology, this mechanism may be particularly relevant in parasitic diseases compared to other chronic infections.

### 3.7 Virtual memory CD8+ T cells in parasitic infections

As noted, parasitic helminths elicit a type 2 immune response characterized by high levels of IL-4. Recent research has found that IL-4 induced by helminth infections activates a special type of CD8+ T cell: virtual memory T cells (TVM). Virtual memory CD8+ T cells are a unique subset of CD8+ T cells found in uninfected mice. Although these cells have never experienced specific antigen stimulation, they are phenotypically and functionally similar to true memory T cells that have experienced the antigen, thus possessing rapid response capability similar to memory T cells (134–136).

Studies showed that helminth infection induces a strong Th2 response, producing large amounts of IL-4, which directly acts on CD8^+^ T cells, driving antigen-independent expansion of virtual memory CD8^+^ T cells without requiring the TCR of CD8^+^ T cells to recognize specific antigens (137, 138). These expanded TVM cells provide broad-spectrum non-specific protection in the event of secondary bacterial infection through a rapid, innate-like response. Even after helminth clearance, TVM cells can remain at high levels for a long period, providing the host with a continuous protective alert state, independent of specific antigen recognition.

Since TVM cells express self-reactive T cell receptors and are therefore exposed to persistent antigenic stimulation, which is a condition known to induce T cell exhaustion, one might expect them to become exhausted. Surprisingly, however, TVM cells do not undergo exhaustion. Studies have shown that UFM1-specific ligase 1 (UFL1), the ligase for ubiquitin-fold modifier 1 (UFM1), promotes TVM cell survival and function by preventing them from acquiring epigenetic, transcriptional, and phenotypic changes similar to those of exhausted T cells, suggesting a key role for UFL1 in inhibiting TVM cell exhaustion. When UFL1 is absent, TVM cells exhibit exhaustion-like characteristics (139).

Another possible anti-exhaustion mechanism of TVM cells involves CD22. During parasitic infections, CD22 inhibits helminth-induced IL-4-driven expansion and activation of self-reactive TVM cells in the periphery, demonstrating its function as a modulator of TVM responses. Conversely, CD22 absence resulted in enhanced TVM responses (140). Therefore, parasitic infection does not lead to TVM cell exhaustion but rather establishes an active “negative regulation” mechanism, which may represent a host balancing strategy to prevent immune system overactivation. This is a prominent characteristic of TVM cells in chronic parasitic infections, quite different from some viral infections. In certain viral infections, such as LCMV infection, TVM cell numbers are rapidly and permanently reduced (>60%), and this reduction results not from migration but from type I interferon-driven selective clearance, which means physical deletion rather than functional “exhaustion” (141). This demonstrates the complex and pathogen-specific influences on TVM cell fate and highlights the unique relationship between helminth infections and this memory-like population.

### 3.8 Myeloid-derived suppressor cells in parasitic infections

Myeloid-derived suppressor cells (MDSCs) are a heterogeneous population of immature myeloid cells derived from the bone marrow that have been arrested for differentiation under pathological conditions (142), including progenitor cells, immature macrophages, immature granulocytes and so on. They are characterized by activation under immunopathological conditions and potent immunosuppressive activity. Although MDSCs were initially discovered in cancer patients (142, 143), increasing research has revealed their crucial role in controlling immune responses to various pathogens. They are a major component of the immunosuppressive network, responsible for suppressing T cell responses under pathological conditions, and are therefore key cells driving T cell exhaustion in chronic parasitic infections.

Parasites, such as Schistosoma eggs and products released by adult worms, continuously stimulate the host, leading to significant MDSC increases in liver, spleen, and peripheral blood (144, 145). MDSCs can secrete arginase-1 (Arg1) and inducible nitric oxide synthase (iNOS), consuming arginine essential for T cell proliferation, and producing toxic reactive nitrogen species that directly interfere with T cell metabolism and function. Furthermore, they suppress immune responses by expressing inhibitory ligands such as PD-L1 and releasing inhibitory cytokines (146–149). This represents a core mechanism leading to T cells’ inability to effectively clear parasites, resulting in an exhausted state.

Additionally, MDSCs, together with Tregs and alternatively activated macrophages (AAMs), construct a robust immunosuppressive network, shaping the immunosuppressive microenvironment that enables parasites to survive long-term within the host. Multiple studies have shown that MDSCs are activated and accumulate in infections caused by *Echinococcus granulosus*, *gastrointestinal nematodes*, *Trypanosoma cruzi*, *Trypanosoma congolense*, *Leishmania donovani*, *Schistosoma japonicum*, and others, playing crucial immunosuppressive roles.

A recent study on chronic *E. multilocularis* infection model revealed that the main reason for the failure of PD-1 blockade therapy is the compensatory accumulation of MDSCs, especially G-MDSCs, around liver lesions, which strongly suppress T cell function through an indoleamine 2,3-dioxygenase 1 (IDO1) - dependent mechanism. Only by combining the elimination of MDSCs can T cell anti-parasitic immunity be significantly restored and parasite growth controlled (87).

Studies on schistosomiasis have also found that interferon regulatory factor 4 (IRF4) regulates MDSCs through the STAT3 and AKT signaling pathways. IRF4 myeloid knockout inhibited the expansion of MDSCs and immunosuppressive function, thereby promoting T cell expansion and enhancing the production of IL-4 and IFN-γ, leading to significantly reduction of liver and lung pathological damage caused by *S. japonicum* infection (150). A clinical study on *S. japonicum* infection showed that G-MDSCs were significantly amplified in the peripheral blood and negatively correlated with T-cell counts, suggesting that they may impair host immune function by suppressing T-cell immunity. Furthermore, G-MDSCs were positively correlated with the degree of liver fibrosis; patients with liver fibrosis had a significantly higher proportion of G-MDSCs than those without fibrosis, confirming the correlation between MDSCs and disease severity (151).

In summary, myeloid-derived suppressor cells are important drivers of T cell exhaustion in chronic parasitic infections. Exploring the interaction network between MDSCs, Tregs, and Th2 cells in parasitic infections may reveal key mechanisms for overcoming T cell exhaustion and enhancing immunotherapy. The prominence of MDSCs in parasitic infections, perhaps even more so than in viral infections, reflects the chronic, granulomatous nature of many parasitic diseases and suggests that MDSC-targeting strategies may be particularly relevant in this context.

## 4 Therapeutic strategy in parasitic infections

In chronic parasitic infections, the key therapeutic targets for T cell exhaustion are primarily focused on immune checkpoints. However, research on reversing T cell exhaustion has evolved from single checkpoint blockade to in-depth analysis of the immunosuppressive microenvironment and the exploration of comprehensive strategies involving multimechanism interventions, such as simultaneously targeting upstream regulatory pathways or combining with depletion of immunosuppressive cells, as illustrated in **Table 1**.

**Table 1:** Therapeutic strategy of parasitic infection.

Therapeutic strategy	Therapeutic target	Mechanism & therapeutic potential	Parasite model/disease	Effect	References
Immune Checkpoint Blockade	CTLA-4	Highly expressed in the marginal zone of lesions in AE patients. In mouse models, anti-CTLA-4 antibody effectively reverses T cell exhaustion, reduces lesion number and weight.	Alveolar echinococcosis (AE)	Significantly reduces lesion number and weight	(152)
	TIGIT	Highly expressed on T cells in the liver and blood of AE patients, positively correlated with lesion activity. In animal studies, blocking TIGIT signaling prevents T cell exhaustion, inhibits disease progression, restores NK cell function, and enhances anti-echinococcal immune responses.	Alveolar echinococcosis (AE)	Successfully prevents T cell exhaustion and inhibits disease progression	(153)
	PD-1	Classic inhibitory receptor; sustained high expression leads to loss of T cell effector function, reduced proliferation, and even apoptosis.	Multiple parasite infections including Toxoplasma gondii, Leishmania spp., malaria, Trypanosoma cruzi	Depends on the complex host-parasite microenvironment. Effective in limiting lesion progression and reducing parasite burden in a mouse model of chronic cutaneous leishmaniasis. However, PD-1 blockade alone may be ineffective or even harmful in other parasite infections.	(68, 83, 87, 154, 155)
	Tim-3	Highly expressed in Toxoplasma-infected mouse models. Anti-Tim-3 antibody treatment reduces tissue cysts by 62% and decreases Tim-3 expression in brain microglial cells.	Toxoplasma gondii, Leishmania mexicana	Effectively reduces parasite burden	(156)
	CD244 (2B4)	Key receptor promoting CD8+ T cell exhaustion. Deficiency of CD244 enhances CD8+ T cell killing function and inhibits parasite growth.	Alveolar echinococcosis (AE)	Effectively inhibits parasite growth	(41)
	LAG-3	Upregulated on exhausted T cells in malaria. In AE, it may regulate CD4+ T cell imbalance and exacerbate disease progression.	Malaria, Alveolar echinococcosis (AE)	Effectively reduce parasite burden and enhance anti-infection immune responses	(157–159)
Immuno- microenvironment Combination Therapy	MDSC (combined with PD-1 blockade)	Depleting MDSCs alone partially restores T cell function, but combining with PD-1 blockade achieves better anti-parasitic efficacy and more effectively activates anti-parasitic immunity.	Alveolar echinococcosis (AE)	Ideal anti-parasitic efficacy in mouse models	(87)
Epigenetic Regulation	PARP1 inhibition	Regulates T cell differentiation and metabolism from an upstream level, promoting CD8+ T cell maturation, stability, and cytotoxicity to control parasite replication.	Trypanosoma cruzi (Chagas disease)	Effectively controls Chagas disease-associated cardiac inflammation	(160)
Vaccine	mRNA vaccine	Lipid nanoparticle mRNA vaccine encoding parasite CD8+ T cell epitopes designed to overcome parasite-mediated T cell dysfunction.	Malaria	Restores and enhances protective immunity	(161)

## 5 T cell exhaustion in other infections

As previously elaborated, T cell exhaustion was initially identified in chronic viral infections, and numerous studies have confirmed T cell exhaustion in various viral models including LCMV, HIV, HCV, HBV, and HTLV-1 (3, 38, 162–164). To date, T cell exhaustion is widely regarded as a general mechanism underlying the tolerization of virus-specific immune responses during chronic infections. Apart from these conventional viral diseases, studies on COVID-19 have also indicated that CD8+ T cell exhaustion may be associated with cytokine storms in severe COVID-19 patients, which resulted in millions of deaths worldwide. A single-cell RNA sequencing data analysis has for the first time demonstrated in severe COVID-19 patients that a cytokine storm promotes CD8+ T cell exhaustion through a specific cytokine-receptor axis, suggesting that targeting the cytokine-receptor axis may be a novel strategy to reverse T cell exhaustion. Related drugs, such as the C-C chemokine receptor 5 (CCR5) antagonist (Maraviroc), are already in clinical trials (165).

T cell exhaustion has also been observed in bacterial infections. In 2015, during a Brucella infection experiment in mice, Durward-Diioia et al. identified a considerable population of exhausted CD8+ T cells expressing LAG-3 and PD-1 that lacked IFN-γ expression, indicating that chronic Brucella infection leads to CD8+ T cell exhaustion, thereby limiting immune function (166). In another study, Mittal et al. found that in the context of pre-existing malignancy, T cell responses to acute bacterial infections exhibit a unique state of phenotypic exhaustion, with significantly elevated expression of inhibitory receptors, suggesting that immune checkpoint therapy targeting inhibitory receptors may have potential value not only in treating tumors but also in treating severe infections in cancer patients (167). Similarly, in a study of Mycobacterium tuberculosis infection (MTB), T cells highly expressed TIM-3 and other inhibitory receptors including PD-1 during chronic MTB infection. Treatment targeting TIM-3 led to reduced bacterial burden, reversing T cell function and improving MTB control (168). PD-1 and its ligand PD-L1 expression is also upregulated in patients with sepsis caused by multidrug-resistant bacteria (169), while BTLA upregulation on lymphocytes and PD-1 upregulation on macrophages both contribute to increased mortality in sepsis (170). The differences in T cell exhaustion between parasitic infections and viral and bacterial infections are shown in **Table 2**.

**Table 2:** Differences in T cell exhaustion between parasitic, viral and bacterial infections.

Feature	Viral infection	Bacterial infection	Parasitic infection
Pathogen nature	Intracellular pathogens, replicate using host cells	Extracellular or facultative intracellular proliferators	Multicellular organisms, with complex life cycles and tissue migration
Antigen dynamics	Persistent high-level antigen (viremia)	Controllable or persistent (e.g., latent tuberculosis)	Antigen fluctuation: different life stages (eggs, larvae, adult worms) release distinct antigens
Dominant immune response	Th1-type	Th1-type	Helminths: Th2-type Protozoa: Th1-type
Features of functional loss	Effector functions are markedly decreased; Proliferative capacity is lost	Characterized by a central loss of CD4^4^ T cell function.	Loss of function is more complex and often accompanied by immunoregulation
Tissue microenvironment	Blood, lymphoid tissues, infected organs	Extracellular spaces, granulomas	Granulomas, cysts (e.g., Echinococcus, Toxoplasma cysts), accompanied by local hypoxia and fibrosis
Key inhibitory receptors	PD-1, LAG-3, TIM-3 (relatively consistent co-expression pattern)	PD-1, CTLA-4, TIM-3	PD-1, LAG-3, TIM-3, TIGIT, CD244, with significant species-dependent differences (e.g., PD-L2 pathway is more critical in filarial infections)
Regulatory cell networks	Tregs and MDSCs involved, but not dominant	MDSCs and Tregs important in chronic phase	Tregs, MDSCs, AAMs (alternatively activated macrophages) highly enriched, forming a complex immunosuppressive microenvironment
Main drivers of exhaustion	Persistent high-antigen stimulation	Persistent antigen + bacterial products	Persistent antigen + parasite-derived immunomodulatory molecules + microenvironmental factors (hypoxia, metabolic competition)
Th1/Th2 imbalance	Th1 persistently activated but functionally impaired	Th1 persistently activated	Helminth infections: Th2 hyper-response with Th1 suppression, and Th2 cells themselves develop hyporesponsiveness (GRAIL-mediated)
Memory formation defect	Impaired memory T cell formation	Partially impaired memory formation	Epigenetic scarring hinders memory formation; long-term protective memory remains difficult to re-establish even after deworming
Temporal dynamics of exhaustion	Can develop rapidly with high antigen load (LCMV model)	Develops during chronic infection phase	Can be extremely delayed (decades in Chagas disease) or develop over months-years
Therapeutic strategies and responses	Anti-PD-1/PD-L1 blockade effective (e.g., LCMV, HIV studies); Combined blockade yields even better efficacy.	Anti-PD-1 in tuberculosis shows variable results	Efficacy varies by parasite species; Anti-TIM-3 shows promise in leishmaniasis and schistosomiasis, but is ineffective or even harmful in echinococcosis and Chagas disease; PD-1 blockade fails in alveolar echinococcosis due to MDSC compensation, requiring combination with MDSC depletion or IDO1 inhibitors

Furthermore, it has been proposed that T cell exhaustion is not exclusive to chronic infections; T cells can also enter an exhaustion-like state in acute viral infections. In an early study in 2015, Erickson et al. found that virus-specific CD8+ T cells isolated from the lungs of mice with acute viral lower respiratory tract infection exhibit a functional state similar to exhausted T cells in chronic infections, highly expressing multiple inhibitory receptors including PD-1, LAG-3, TIM-3 and so on (171). And their transcriptional profiles significantly overlap with those of exhausted CD8+ T cells in chronic LCMV infection, rather than with effector/memory T cells. Similar functional states may also exist in acute central nervous system infections, such as those observed during rabies and coronavirus infections (172, 173). A recent study in 2025 has also confirmed it. Chu et al. demonstrated that progenitor exhausted T cells (Tpex) are generated during acute infections and in the early infection phase, long before infection becomes resolved or chronic (174), supporting the view exhaustion programs can be initiated early, even in infections that will ultimately resolve, suggesting that T cell differentiation may follow a common pathway, with the eventual outcome (memory versus exhaustion) determined by the duration and intensity of signals received during this critical early window. However, whether such early Tpex formation occurs in acute parasitic infections (e.g., acute toxoplasmosis) and how it might influence subsequent chronic stages remains an open question that warrants further investigation.

## 6 Conclusion

Up till the time that this study was carried out, numerous studies have investigated the mechanisms and development of T cell exhaustion. The occurrence and progression of T cell exhaustion represent a complex process involving multiple interacting factors, regulated by persistent antigen exposure, inhibitory receptors, soluble cytokines, metabolic microenvironment, and other elements. Alterations in any of these factors may compromise the body’s ability to effectively control infections or tumors.

In response to these challenges, related animal experiments and clinical studies have revealed new possibilities for immune-targeted therapy. For example, reversing T cell exhaustion by targeting inhibitory pathways on exhausted T cells through checkpoint blockade during chronic viral infection and cancer has remained a focal topic in immunotherapy research.

However, research on exhausted T cells has predominantly focused on viral infections and tumors. In parasitic infections, the study of T cell exhaustion remains insufficient. This knowledge gap is particularly significant because parasitic infections, with their complex life cycles, chronic course, tissue-specific pathology, and Th2-biased or immunoregulatory environments, may engage distinct exhaustion mechanisms that are not captured by viral or tumor models. Key questions remain unanswered: How do helminth-induced Th2 responses interface with exhaustion programs? What role do parasite-driven tissue hypoxia and granuloma formation play in local T cell dysfunction? Can the unique resistance of virtual memory T cells to exhaustion be therapeutically harnessed? Do the distinct temporal dynamics of exhaustion in parasitic infections (e.g., decades in Chagas disease) offer a wider window for intervention?

The state of T cell exhaustion in infections, particularly in non-viral contexts, requires extensive further study. Understanding how different classes of pathogens shape T cell differentiation and dysfunction will not only illuminate fundamental immunology but also guide the rational development of immunotherapies for the full spectrum of chronic infectious diseases.
